# Ameliorating quality and vase life of *Solidago canadensis* flowers via supplementation of eucalyptus, neem and rosemary as phyto-preserver oils

**DOI:** 10.1186/s12870-025-07131-3

**Published:** 2025-08-14

**Authors:** Iman Mohamed El-Sayed, Rasha Ahmed Mohamed El-Ziat, Hani Saber Saudy, Mohammed Hewidy

**Affiliations:** 1 Ornamental Plants and Woody Trees Department, National Research Centre (NRC), Dokki, Cairo Egypt; 2 Ornamental Horticulture Department, Faculty of Agriculture, Cairo University, Giza, Egypt; 3 Agronomy Department, Faculty of Agriculture, Ain Shams University, 68- Hadayek Shoubra, Cairo, 11241 Egypt; 4 Horticulture Department, Faculty of Agriculture, Ain Shams University, 68- Hadayek Shoubra, Cairo, 11241 Egypt

**Keywords:** Flower longevity, Microbial infection, Natural oils, Ornamentals, Solidago quality

## Abstract

**Purpose:**

The loss of flower quality after harvesting is a major concern in the floriculture industry. Because cut flower solutions are quickly contaminated with microbes, causing flower damage, they must be modified to enhance and extend the life of the vase.

**Methods:**

Eco-friendly preservative solutions were examined to investigate the efficiency of natural essential oils of eucalyptus, neem and rosemary at concentrations of 200 and 400 mg L^–1^ each on the biological, physiological, and anatomical traits and vase life of solidago (*Solidago canadensis*) cut flower.

**Results:**

Using different essential oils at both concentrations showed significant impact on cut flower longevity. The maximum vase life was obtained by solidago placed in a preservative solution containing 400 mg L^–1^ of neem essential oil, which enhanced water uptake and relative fresh weight and reduced both water loss and microbial count when compared to other treatments and control treatment. The chlorophyll, total phenol, flavonoid, and carotenoid content of the spikes increased in solidago cut-flower placed in a preservative solution containing neem essential oil 400 mg L^−1^. Results also showed decreased malondialdehyde (MDA), hydrogen peroxide (H_2_O_2_) levels and total antioxidant activity (DPPH radical-scavenging activity) with the application of all natural oils supply. Anatomically, cut flowers that treated with essential oils had considerably clearer vessels and significantly fewer bacteria than untreated cut flowers.

**Conclusion:**

The higher concentration of different essential oils gave better results than the lower concentration. These results suggest that for the floriculture industry, natural phyto-oils provide a clear viable method to extend the vase life of solidago cut flowers. Thus, neem oil at a concentration of 400 mg L^–1^ added to a preservative solution is considered the most promising practice for prolonging the vase life and maintaining high quality of Solidago.

## Introduction

The cultivation of ornamental plants is a growing sector globally, with great potential for future growth in international markets [1–4]. Unfortunately, the short vase life of cut flowers, which is impacted by both environmental and genetic factors, frequently inhibits future development [5–7]. In the *Asteraceae* family, the genus *Solidago* is well known, in a commercial sense as a provider of cut flowers. *Solidago canadensis* denotes one of the oldest ornamental plants originating from North America to Europe [8]– [9]. In the last century, solidago flowers prevailed all over Europe [10] and in Asia [11–13]. They also spread in Africa [14] and Australia [15]. Moreover, it has been identified for years as a landscape plant [16]– [17]. Solidago is commonly used as a fabulous cut flower for indoor decoration in vases and bowls [18]. In addition to its medicinal usefulness, solidago is listed as a first-class alternative source for floriculture businesses [19].

Cut flower’s shelf life after harvesting is frequently affected by clogging of microbes, stem occlusion or physiological occlusion, preventing the absorption of water or extracellular enzymes that can damage the walls of vascular tube-cell [20]– [21]. To resolve this problem and expand the shelf-life of cut-flowers, it is more advisable to use natural substances [20, 22–24] that have no adverse effects on human health and the environment (like essential oils) and which are fairly cheap since most chemicals used for the same purpose are toxic and they can cause environmental pollution [22, 25, 26]. Herbal extracts and essential oils have antimicrobial properties and are used for their ability to prolong the lifespan of cut flowers. Essential oils are natural products or organic that obtained from medicinal and aromatic plants [23]. They could be used as preservative solutions to control bacterial and fungal pathogens [10].

Gum tree (*Eucalyptus* spp.) is a tree belonging to the family Myrtaceae with special active ingredients that showed wide range of applications [27]. The essential oils of *Eucalyptus* have many medicinal and commercial uses [28]. The main components of *Eucalyptus* oil are anodyne, anaesthetic, antiperiodic, antiseptic, antiphlogistic, astringent, diaphoretic, deodorant, disinfectant, febrifuge, expectorant, fumigant, hemostat, insect repellant, inhalant, preventative, sedative yet stimulant, rubefacient, suppurative, vermifuge and tonic [27, 29–31].

Neem (*Azadirachta indica*) is a tree in the Meliaceae family [32]. A complex tetranortriterpenoid limonoid is the main constituent of the essential oil of neem, which has a toxic effect on insects [33]. The essential oils of neem have various anti-bacterial and antimicrobial properties against some pathogens due to polyphenolic flavonoids (nimbanene, nimbin, nimbandiol, 6-desaacetylnimbinene, ascorbic acid, nimbolide, amino acid, 7-desaacetyl-7-benzoylgedunin, n-hexacosanol,7-desaacetyl-7-benzoylazadira-dione, 17-hydroxyazadiradione, and Beta-sitosterol nimbiol Quercetin [34]. Earlier [35], found out that using the holding solutions with neem extract added at 2.0% led to increased vase life of the solidago cultivar Tara.

Rosemary (*Rosmarinus officinalis* L.) belongs to Lamiaceae family. The main component of rosemary is a volatile oil which is rich in phenolic compounds [21]. Also, the essential oil of rosemary contains various pinene, antioxidant components, and camphor which are associated mainly with the classes of flavonoids, phenolic acids, and diterpenoids [18, 36]. Basiri et al. [37] found that the vase life of flowers improves to 24 days with using rosemary extract and sucrose in the preservative solution. Also [14], indicated that the rosemary essential oil improves the vase life of the Alstroemeria flower.

However, there is limited information or no reports about using essential oils as natural floral preservative to improve postharvest parameters and the quality of solidago cut flowers or any cut flowers preserving solution. Therefore, the current study hypothesized the natural phyto-oils could ameliorate quality and vase life of solidago flowers via altering physiological and anatomical features. Herein, this study aimed to estimate the efficiency of essential oils from gum tree, neem and rosemary, individually, with different concentrations in floral preservatives to increase longevity, enhance chemical composition, and reduce bacterial growth in floral preservatives of cut solidago flowers as well as to find the most effective, eco-friendly, sustainable and affordable floral preservative.

## Materials and methods

### Plant material

The fresh flowers of solidago (*Solidago canadensis*) were obtained from Floramax, a commercial Farm in Kafr Hakim, Giza, in two successive seasons 2022–2023. The flowers were harvested in the early morning when 25% of top flowers of the inflorescences were opened, then immediately transported to the laboratory of the Ornamental Horticulture Dep., Fac. of Agric., Cairo Univ., Giza, Egypt. To counteract the effect of high temperature in the field, the cut flowers were placed at 4 °C for 1-h. After cutting the stems of solidago flowers to a length of 60 cm underwater, the flowers were soaked in cold water for 30 min. Additionally, the leaves on the lower third of the stem were removed keeping 25 ± 2 leaves. Essential oils were obtained from the unity of squeezing and extracting natural oils, National Research Centre (NRC) where they were obtained from Eucalyptus leaves, neem seeds and rosemary herb. Under a relative humidity of 65% and a 12-hour light/12-hour dark photoperiod, only one flower was placed in the vase (1000 ml) containing a vase solution of 600 ml of preservation solution treatments including (eucalyptus, neem and rosemary oils) 22 ± 2 °C.

### Treatments of essential oils

Essential oils were supplied in the form of a holding solution containing 2.0% sucrose. Three different essential oils were used, which are obtained from eucalyptus leaves (EU), neem seeds (NM) and rosemary herb (RO) dissolved in Tween-80 (0.1% v/v) + 10% ethyl alcohol. Different holding solutions were made up in distilled water at concentrations of 200 and 400 mg/L of each essential oils in addition to the control treatment (CK) that was kept in the same condition with only distilled water. Thus, the treatments involved EU with 200 and 400 mg/L (EU200 and EU400), NM with 200 and 400 mg/L (NM200 and NM400), RO with 200 and 400 mg/L (RO200 and RO400) and control.

### Data recorded

#### Floral performances

##### Vase life

**Vase life (days):** The longevity of solidago flowers in a vase was determined by observing the number of days from cutting until the petals showed signs of senescence, petal loss, and discoloration [38].

**Vase solution uptake (g/day**): It was recorded per single flower as the weight of absorbed vase preservative solution (g) for each treatment at 3, 5, and 7 days, according to Bazaz et al. [39].

**Relative fresh weight** (%): At the beginning of the experiment, we measured the weight of solidago flowers. Subsequently, we recorded the weight on days 3, 5 and 7 during the vase life period. Cut flower’s relative fresh weight (RFW%) = (Wt/W t^0^) × 100; adapted from He et al. [40], where Wt is flowers weight (g) at 0, 3, 5, 7 days W t^0^ is weight of the same flowers (g) at starting date of vase.

**Water loss (g/day**): Throughout the experiment time, water loss was determined as the weight of vase solution and cut flowers. At 3, 5 and 7 days of each treatment during vase life period water loss was recorded per single flower as follows: Water loss (g/day) = (Ct^−1^-Ct); where Ct is the weight of vase solution and cut flowers at 3, 5 and 7 days, and Ct^−1^ is the coapplied of the weight of vase solution and cut flowers on 0, 3, 5, and 7 days [39].

##### Photosynthetic pigments

The content of chlorophyll a, chlorophyll b, total chlorophyll and total carotenoids was analysed in the leaves after seven days within the shelf-life period according to Saric et al. [41].

##### Secondary metabolites

Total phenols in dried leaves were determined using Folin–Ciocalteu’s reagent according to the method reported by Swain et al. [42]. Measurements were performed with a spectrophotometer (Model SM1200; Randolph, NJ, USA) was used. Total flavonoids were quantified using a colorimetric assay in dried leaves following Quettier et al. [43].

#### Oxidative stress indicators

Total antioxidant activity (DPPH) was measured in terms of hydrogen-donating or radical-scavenging ability by using the stable radical DPPH according to Brand-Williams et al. [44]. The samples of fresh florets were taken after 7 days during the vase-life period to quantify hydrogen peroxide (H_2_O_2_). Oxidative stress markers, including H₂O₂, in fresh florets were measured at 390 nm using a spectrophotometer (Unico UV-2100, USA) according to the method proposed by Patterson et al. [45]. According to Heath and Packer [46], the Malondialdehyde (MDA) concentration was analyzed to determine the amount of lipid peroxidation. A mixture of 0.5 g of fresh leaves and 5.0 mL of trichloroacetic acid (TCA) (5% w/v) was combined, and the mixture was centrifuged at 12,000 g for 10 min at 4 ˚C. Two mL of the extract and two mL of 0.6% thiobarbituric acid (TBA) were mixed and heated to 95 ˚C in a water bath for ten minutes. At 532 and 600 nm, absorbance was measured. The following formula was used for MDA calculation: MDA content (mmol kg–1) = 6.45 × (A532 − A600) − 0.56 × A450.

### Bacterial count

Bacterial abundance in preservative solutions was quantified via colony-forming unit (CFU) enumeration using the plate count method [47]. A volume of approximately one mL was extracted from every treatment sample. Sterilized distilled water was used to dilute the distilled water (control) from the first to the sixth dilution. After that, Petri dishes with media containing agar, peptone, and beef extract were inoculated with 1 ml of each of the fourth, fifth, and sixth dilutions using a calibrated pipette. After pouring and inoculation, the plates were placed in a calibrated and certified incubator operating under sterile, controlled environmental conditions. Incubation was maintained at 35 ± 0.5 °C for 48 h. Incubation complete: After 48 ± 3 h at 35 ± 0.5 °C, plates were removed and allowed to cool to room temperature inside the biosafety cabinet. Plates containing between 20 and 300 discrete colonies were deemed countable. Plates outside this range were discarded or noted as “TFTC” (too few to count) or “TNTC” (too numerous to count). Using a sterile, magnifying hand-counter, each colony was marked and tallied and verification for quality assurance. Colony counts (CC) were converted to concentration colony forming units per mL (CFU/mL) by applying the appropriate dilution factor (D): CFU/mL = CC/1mL*D, Where D is the reciprocal of the sample dilution. The mean CFU/mL across replicates was reported and statistically analysed.

### Scanning electron microscopy (SEM)

The xylem was scanned by using a SEM - JEOL JSM-6390. First, at the end of the shelf life, 0.5 cm cut parts of the segments of xylem were aseptically excised from all treatments and the control using a sterilized scalpel. Longitudinal sections of the stem were made at multiple levels. Each segment was affixed, face up, to a conductive adhesive tab on an aluminum SEM stub using conductive carbon glue. Mounted sections were sputter coated with a thin layer (≈ 10 nm) of gold to render the surface conductive, evacuation and charging under the electron beam [48].

### Experimental design and statistical analysis

During the two experimental seasons (2022 and 2023), three replicates of each treatment were used in a randomized complete design, solidago flowers were single spikes in each vase (1000 ml) as replicate contains 5 biological replicates (vase) per treatment. Data were analysed using a one-way ANOVA test, and novel multiple-range tests were used to assess the treatments’ average for significance at the 0.05% probability (*p* < 0.05) level [49]. For generating the Pearson’s correlation as heat-map and principal component analysis (PCA) biplot-building, the IBM SPSS (version-25, SPSS Inc., Chicago, IL, USA) and R (version 4.0.2) and statistical software programs were utilized.

## Results

### Floral performances

#### Vase life

**Fig. 1:**
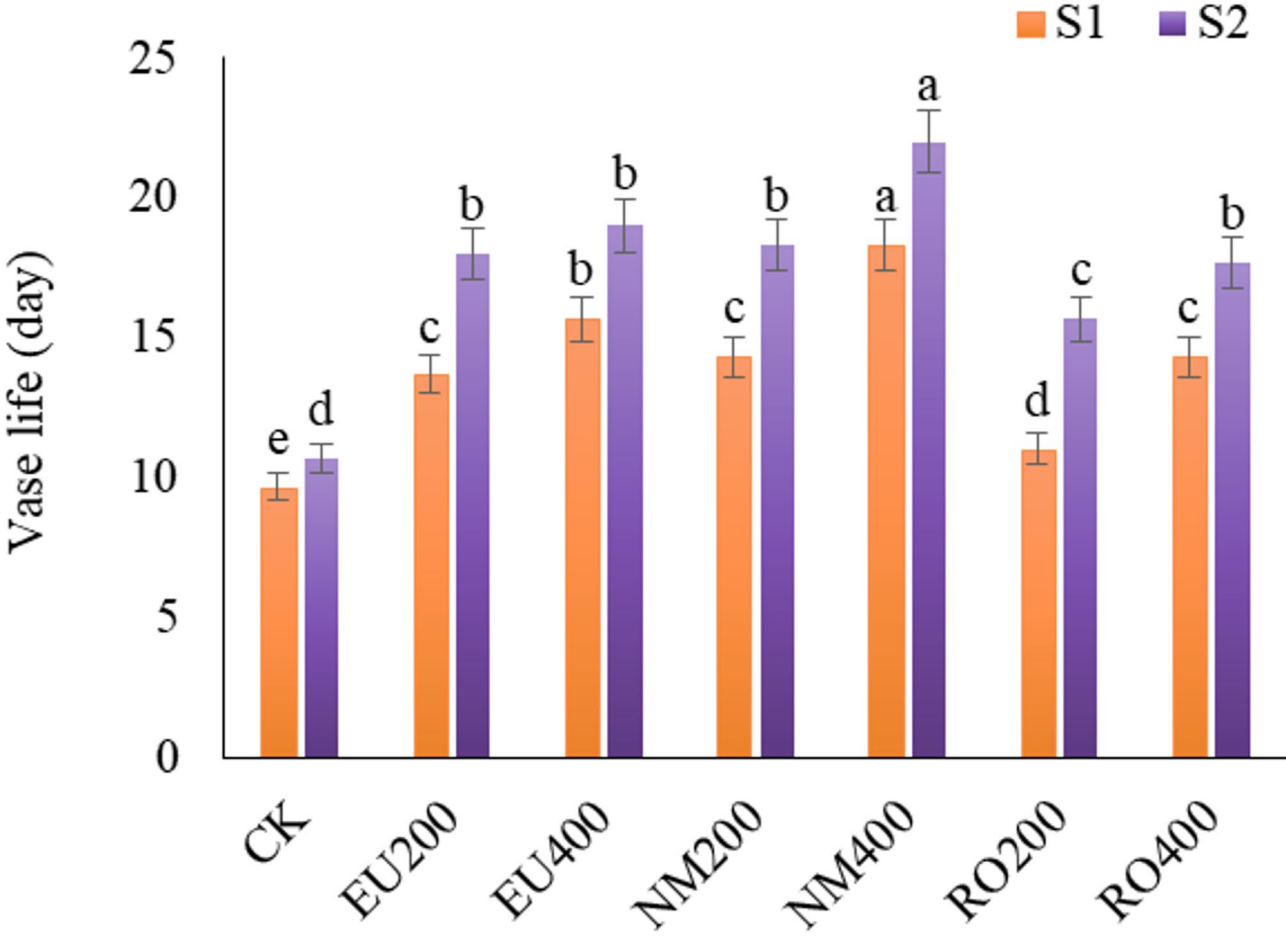
Response of *Solidago canadensis* cut-flowers vase life to natural essential oils as preservative solutions in 2022 (S1) and 2023 (S2) seasons. EU200 and EU400: Eucalyptus oil at 200 and 400 mg L^–1^; NM200 and NM400: Neem oil at 200 and 400 mg L^–1^; RO200 and RO400: Rosemary oil at 200 and 400 mg L^–1^, respectively; CK: control treatment. Bars with different letters are statistically significant at 0.05 level of probability (*p* < 0.05)

**Fig. 2:**
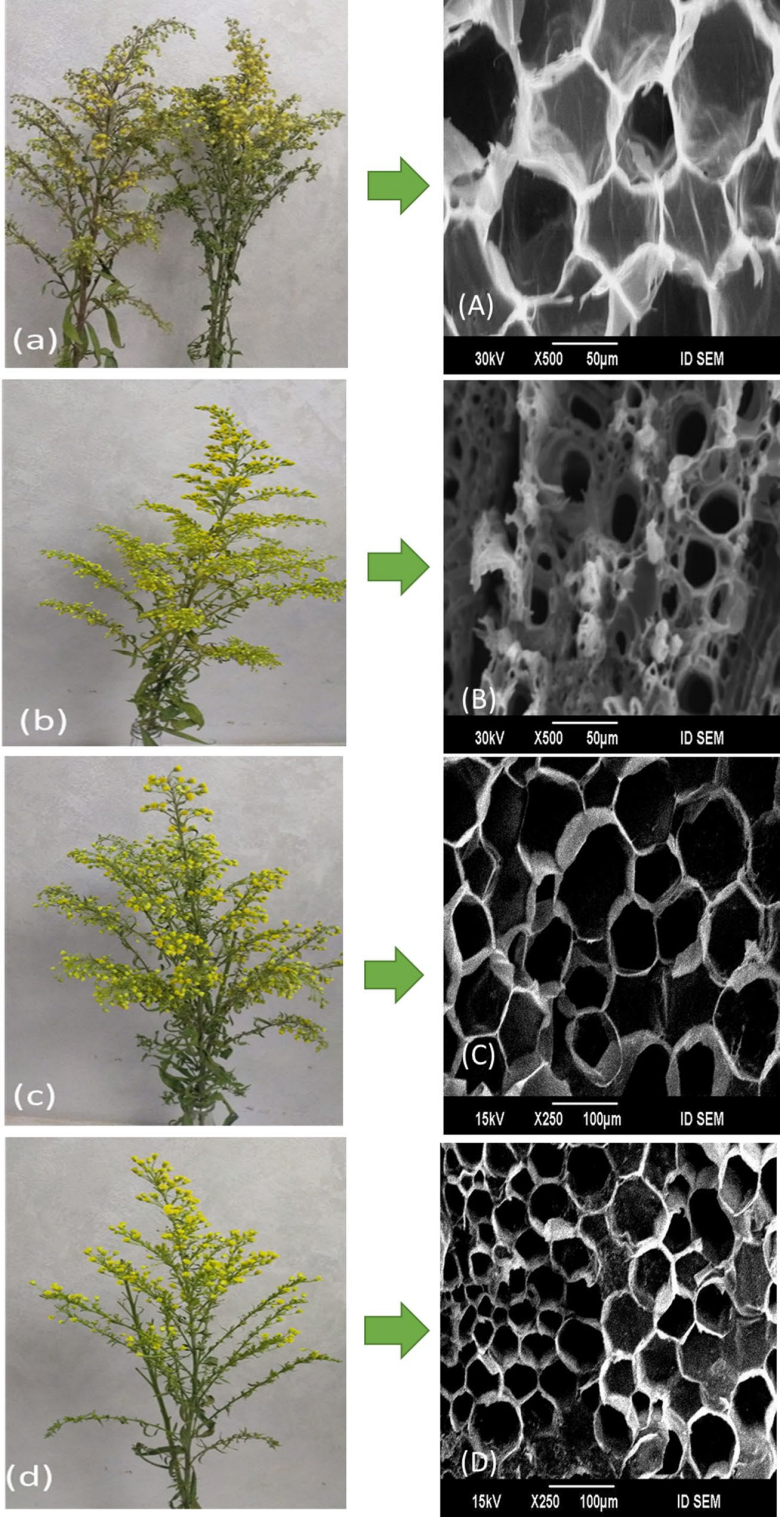
Photo-illustrations showing the effect of essential oils on *Solidago canadensis* cut-flower vase life appearance after 9 Days. a, **A** Control treatment; b, **B** Eucalyptus oil at 400 mg L^–1^; c, **C** Neem oil at 400 mg L^–1^; d, **D** Rosemary oil at 400 mg L^–1^

Result data illustrated in Fig. 1 indicate that the addition of various types and concentrations of essential oils gave longer vase life with significantly (*p* < 0.05) differences than control treatment. Neem oil in the holding solution was the most effective in extending the vase-life of solidago cut-flowers. The highest mean of vase-life (day) was realized using the holding solution containing 400 mg/L of Neem oil (NM400), which recorded 18.33 and 22 days, in respect order in the first and second seasons, outperforming the control treatment by 1.9 and 2.1 folds, respectively. All essential oils applied showed that the decrease in the level of essential oils led to a small decline in the vase life of the cut-flowers, even though they are still more effective than untreated cut-flowers. The vase life of solidago was extended by higher concentrations of different essential oils applied, while the untreated and lower levels of essential oils showed a decrease in vase life and early loss of visible attributes (Fig. 2).

### Relative fresh weight

**Fig. 3:**
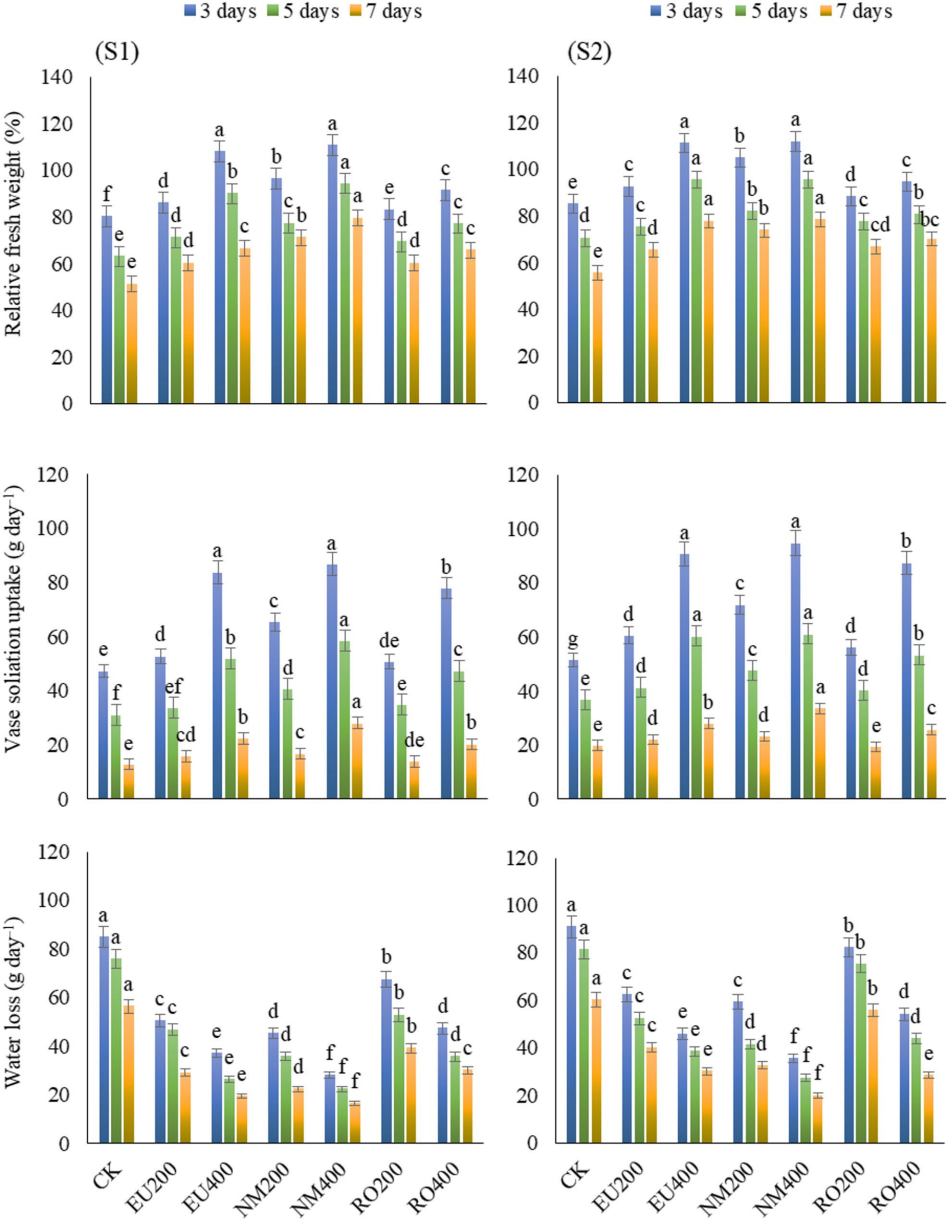
Influence of natural essential oils as preservative solutions on relative fresh weight, vase solution uptake, and water loss at day 3, 5 and 7 of vase life period in *Solidago canadensis* cut-flowers in 2022 (S1) and 2023 (S2) seasons. EU200 and EU400: Eucalyptus oil at 200 and 400 mg L^–1^; NM200 and NM400: Neem oil at 200 and 400 mg L^–1^; RO200 and RO400: Rosemary oil at 200 and 400 mg L^–1^, respectively; CK: control treatment. Bars with different letters are statistically significant at 0.05 level of probability (*p* < 0.05)

According to the result data displayed in Fig. 3, there were significant (*p* < 0.05) differences in relative fresh weight (RFW) of treated cut-flowers observed after 3, 5 and 7 days from starting the experiment. RFW of NM400 treatment was higher than the other treatments from day 3 up to 5 and 7 days in both seasons. However, EU400 showed a similar result to the one observed by NM400 in the 2nd season.

### Vase solution uptake

Result data illustrated in Fig. 3, show quite clearly that there was a significant (*p* < 0.05) difference in vase solution uptake (VSU) of treated solidago cut-flowers observed after 3, 5 and 7 days from starting the experiment. The increase in concentration of the essential oils led to a significant increase in VSU. Cut-flower VSU of NM400 treatment was higher than other treatments after 3, 5 and 7 days in both seasons. VSU increased from 47.3 to 51.4 mL in untreated cut-flowers to 86.7 and 94.7 mL in NM400-treated ones after 3 days in respect order of the two seasons. VSU amount was reduced with the standing of cut-flowers for a longer time which reached 12.7 and 19.8 mL in untreated flowers to 28.0 and 33.5 mL in NM400-treated ones after 7 days in respect order of seasons. Moreover, the values of VSU RO200 after 3 day in the first season and after 7 days in both seasons were as similar as the control treatment.

### Water loss

As illustrated in Fig. 3, result data demonstrate that there was a significant (*p* < 0.05) difference in water loss of treated cut-flowers observed after 3, 5 and 7 days from starting the experiment. The increase in essential oils concentration led to a significant decrease in water loss. NM400 showed the maximum significant reductions in water loss outperforming the control treatment and all other essential oil applications after 3, 5 and 7 days of the experiment beginning in both seasons.

### Photosynthetic pigments

**Fig. 4:**
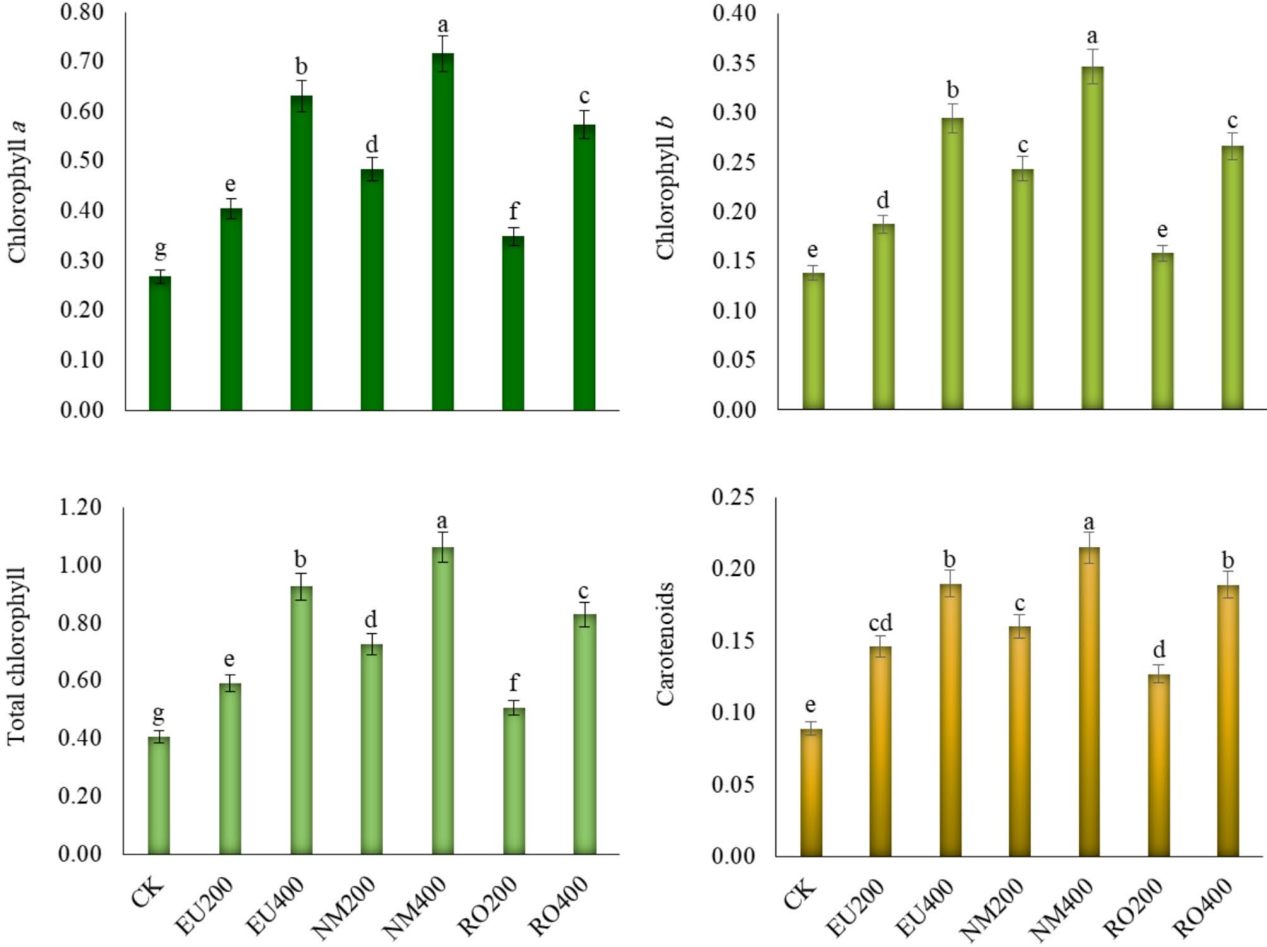
Influence of natural essential oils as preservative solutions on chlorophyll *a*, chlorophyll *b*, total chlorophyll and carotenoids (mg g^–1^ fresh weight) in *Solidago canadensis* cut-flowers. EU200 and EU400: Eucalyptus oil at 200 and 400 mg L^–1^; NM200 and NM400: Neem oil at 200 and 400 mg L^–1^; RO200 and RO400: Rosemary oil at 200 and 400 mg L^–1^, respectively; CK: control treatment. Bars with different letters are statistically significant at 0.05 level of probability (*p* < 0.05)

Chlorophyll *a*, chlorophyll *b*, total chlorophyll and carotenoids concentrations showed significant (*p* < 0.05) differences among essential oil treatments (Fig. 4**)**. All essential oil treatments were significantly (*p* < 0.05) higher in all pigments concentrations than the control treatment, except RO200 for chlorophyll *b*. Also, the higher essential oil concentration had a positive significant (*p* < 0.05) effect compared to the lower concentration. The highest values of all pigments belonged to NM400 treatment with a higher significant (*p* < 0.05) value compared to other treatments, exhibiting increase of 2.7, 2.5, 2.6 and 2.4 times greater than the control treatment.

### Secondary metabolites

**Fig. 5:**
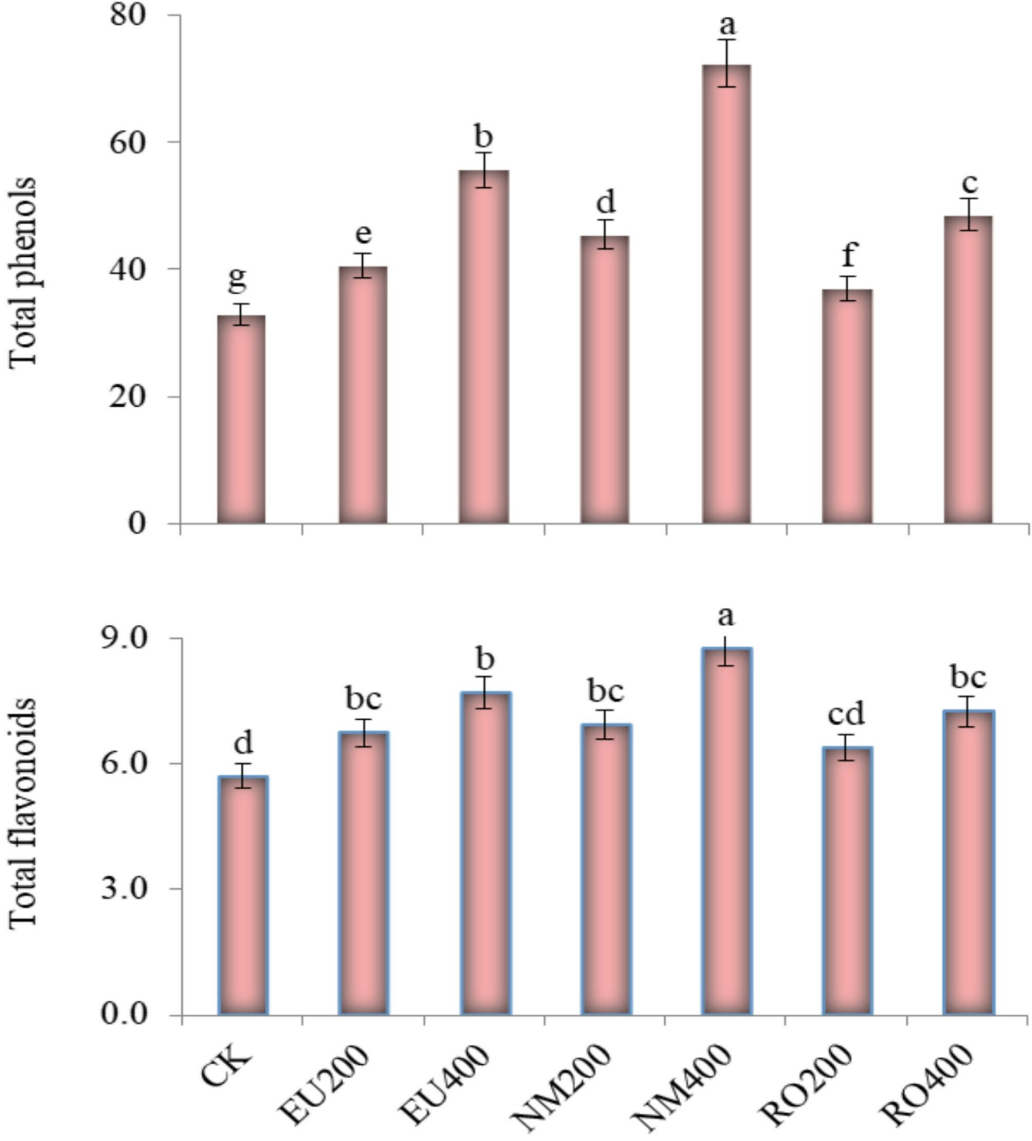
Influence of natural essential oils as preservative solutions on total phenols and total flavonoids (mg g^–1^ fresh weight) in *Solidago canadensis* cut-flowers. EU200 and EU400: Eucalyptus oil at 200 and 400 mg L^–1^; NM200 and NM400: Neem oil at 200 and 400 mg L^–1^; RO200 and RO400: Rosemary oil at 200 and 400 mg L^–1^, respectively; CK: control treatment. Bars with different letters are statistically significant at 0.05 level of probability (*p* < 0.05)

The use of different essential oils at both concentrations significantly (*p* < 0.05) increased total phenols and total flavonoid contents when compared to control treatment. The use of NM400 increased significantly (*p* < 0.05) total phenol and total flavonoid contents greater than the other remaining treatments (Fig. 5). Total phenol and total flavonoid contents increased in all treatments at higher essential oil concentrations when compared to lower concentrations. Still, lower essential oil concentrations were connected with higher phenol and total flavonoid contents when compared to the control treatment.

### Oxidative stress indicators

**Fig. 6:**
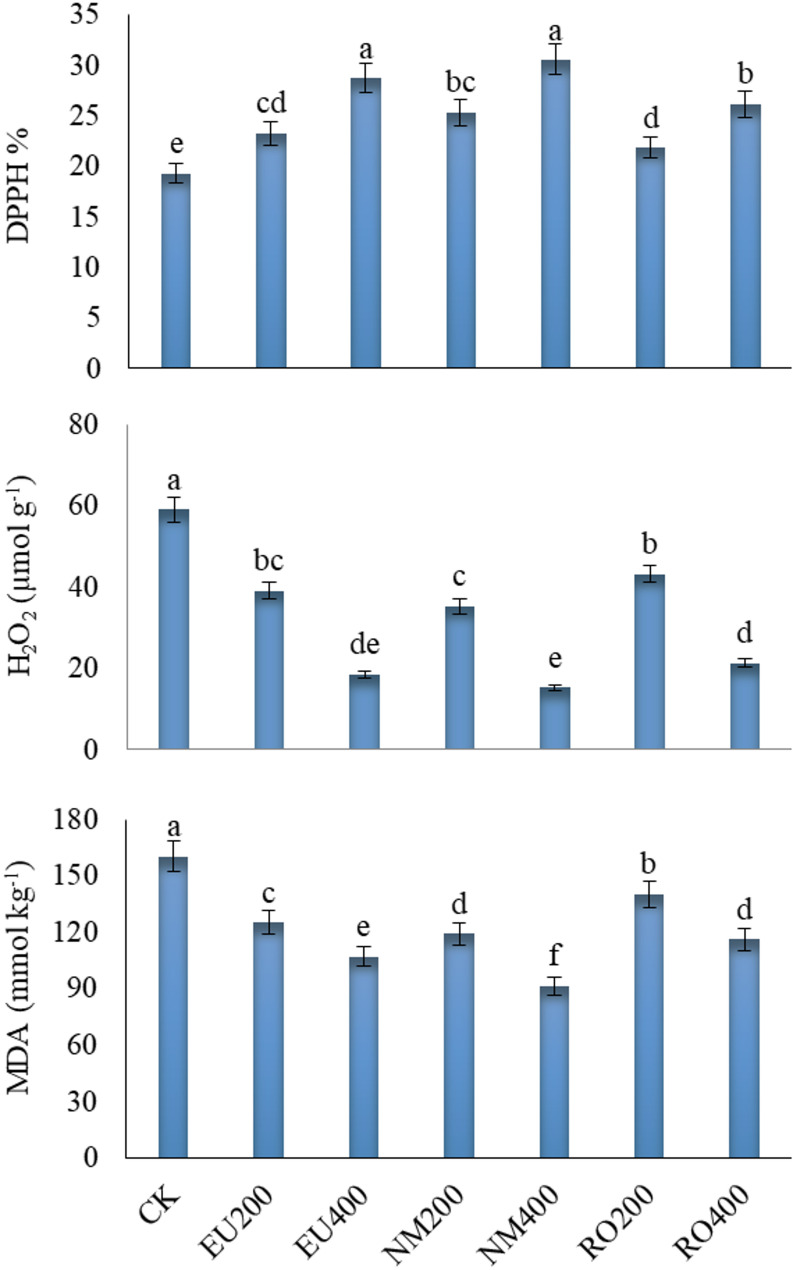
Influence of natural essential oils as preservative solutions on total antioxidant activity (DPPH), malondialdehyde (MDA) and hydrogen peroxide (H_2_O_2_) in *Solidago canadensis* cut-flowers. EU200 and EU400: Eucalyptus oil at 200 and 400 mg L^–1^; NM200 and NM400: Neem oil at 200 and 400 mg L^–1^; RO200 and RO400: Rosemary oil at 200 and 400 mg L^–1^, respectively; CK: control treatment. Bars with different letters are statistically significant at 0.05 level of probability (*p* < 0.05)

The highest significant (*p* < 0.05) value of DPPH was observed in leaves of cut-flowers treated with NM400 along with EU400 outperforming the control treatment and other oil treatments (Fig. 6). Such two treatments increased DPPH by 58.6 and 49.3%, respectively, grater that the control treatment. The other essential oil applications and their concentrations surpassed the control treatment for increasing DPPH. Conversely, all essential oil additions reduced the **H**_**2**_**O**_**2**_ and MDA (Fig. 6) compared to the control treatment. Herein, the maximum reductions were obtained with NM400 treatment, amounting to − 74.2% for **H**_**2**_**O**_**2**_ and − 43.0% for MDA, compared to the control treatment.

### Bacterial counting

**Fig. 7:**
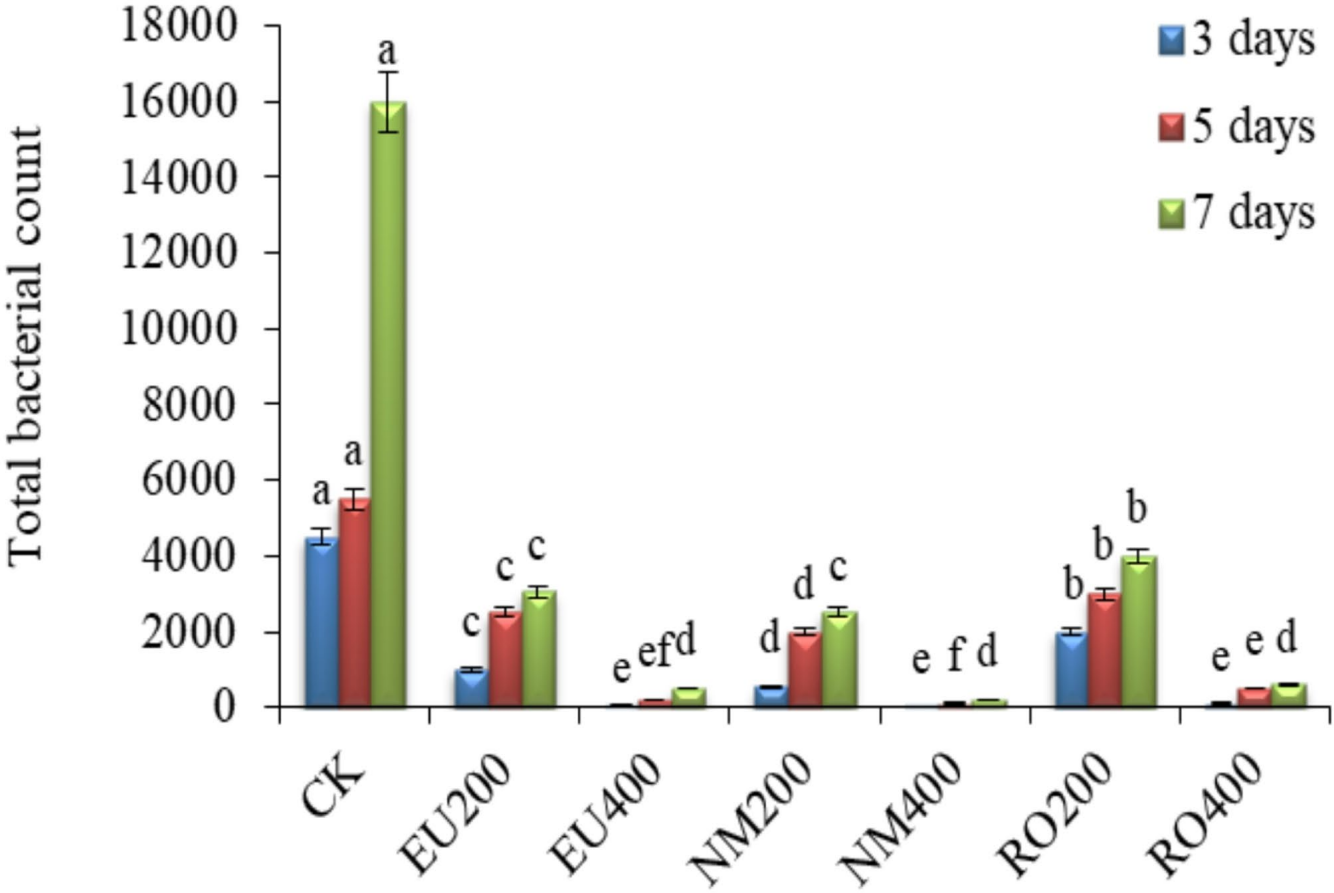
Influence of natural essential oils as preservative solutions on total bacterial count (C.F.U ml^–1^) in growth medium at day 3, 5 and 7 of vase life period of *Solidago canadensis* cut-flowers. EU200 and EU400: Eucalyptus oil at 200 and 400 mg L^–1^; NM200 and NM400: Neem oil at 200 and 400 mg L^–1^; RO200 and RO400: Rosemary oil at 200 and 400 mg L^–1^, respectively; CK: control treatment. Bars with different letters are statistically significant at 0.05 level of probability (*p* < 0.05)

The microbial population increased with extending cut-flowers by the time of preserved and untreated cut-flowers, with the highest values in the control treatment (Fig. 7). Essential oil application significantly reduced number of bacteria compared with the control treatment. Reduction was more noticeable with the higher concentration of all essential oil types. So, the essential oils of eucalyptus, neem, and rosemary at the higher concentration were the most successful in inhibiting bacteria and fungi growth compared to untreated cut-flowers.

### Observation of scanning electron microscopy (SEM)

**Fig. 8:**
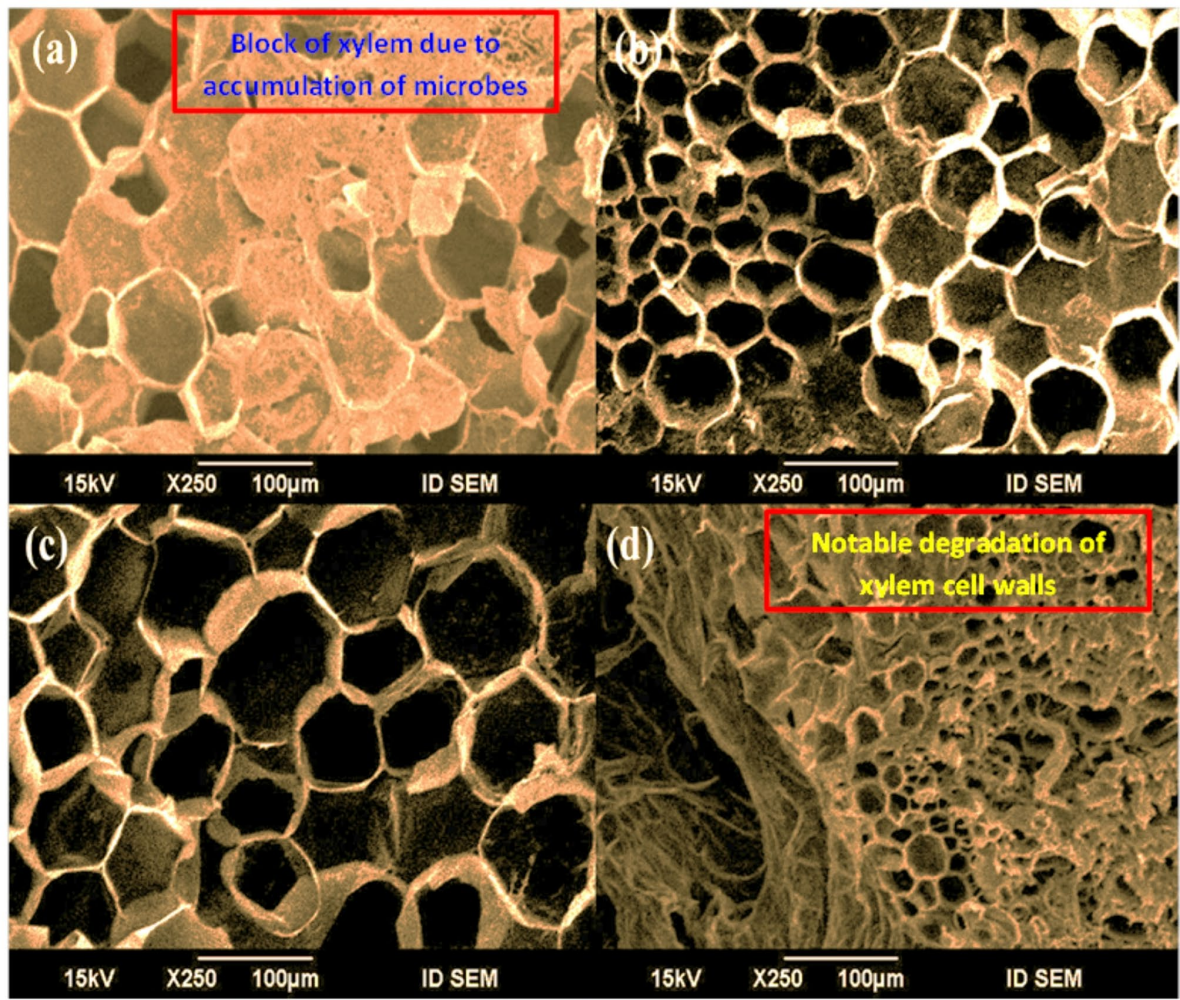
Cross section at the base stem of *Solidago canadensis* cut-flower. The sections were made at the end of shelf life after (10 days). **a** Control treatment; **b** Eucalyptus oil at 400 mg L^–1^; **c** Neem oil at 400 mg L^–1^; **d** Rosemary oil at 400 mg L^–1^

SEM images of the stem bases of cut-flowers were collected from cut-flowers treated and untreated with essential oils observing the morphological characteristics of xylem cells (Fig. 8). Cut-flowers that were treated with essential oils had considerably clearer vessels and significantly fewer bacteria and less degenerative effects due to wound healing process than cut-flowers that were untreated, which had obstructions and bacteria accumulation in their xylem vessels. In the control treated cut-flowers, xylem blocking and degradation of cell walls occurs by microorganisms and their exudates at the floral stem end (Fig. 8a). Cut-flower xylem vessels were not closed with the wound healing impact on cell walls at the turgor level when treated with NM400 (Fig. 8c).

### Correlation coefficient

**Fig. 9:**
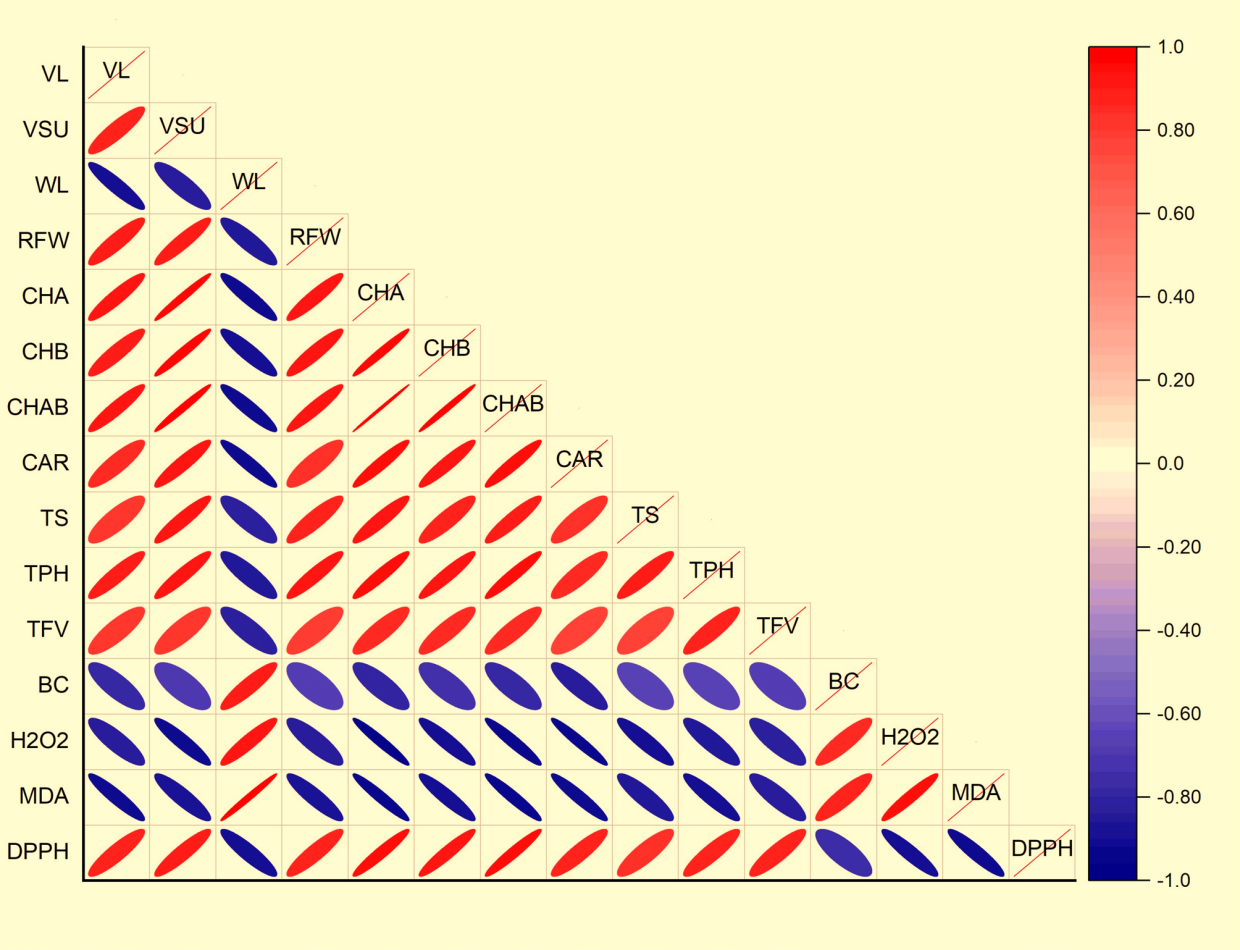
Heat-map shows the analysis of correlation among different solidago cut-flower traits. VL: vase life, VSU: vase solution uptake, WL: water loss, RFW: relative fresh weight, CHA: chlorophyll a, CHB: chlorophyll b, CHAB: chlorophyll ab, CAR: carotenoids, TS: total soluble sugar, TPH: total phenol, TFV: total flavonoids, BC: bacterial count, H_2_O_2_: Hydrogen peroxide, MDA: Malondialdehyde

The correlation coefficients among studied parameters of physiological, chemical and microbial count of treated cut-flower of solidago are presented in a heat map (Fig. 9). There was a notable high significant (*p* < 0.05) positive correlation obtained between vase life and each of vase solution uptake, relative fresh weight, chlorophyll *a*, chlorophyll *b*, total chlorophyll, carotenoids, total soluble sugar, total phenol, total flavonoids and negative correlations with bacterial count, hydrogen peroxide, malondialdehyde. Furthermore, highly significant (*p* < 0.01) and negative correlations were observed with water loss and each of vase solution uptake, relative fresh weight, chlorophyll *a*, chlorophyll *b*, total chlorophyll, carotenoids, total soluble sugar, total phenol, total flavonoids and negative correlations with bacterial count, hydrogen peroxide, malondialdehyde. On the other hand, the bacterial count showed a highly significant (*p* < 0.01) and negative correlation with the most important traits that may affect flower turgor, pigment and antioxidant properties each of vase solution uptake, relative fresh weight, chlorophyll *a*, chlorophyll *b*, total chlorophyll, carotenoids, total soluble sugar, total phenol, total flavonoids and DPPH.

### Principal component analysis (PCA)

**Fig. 10:**
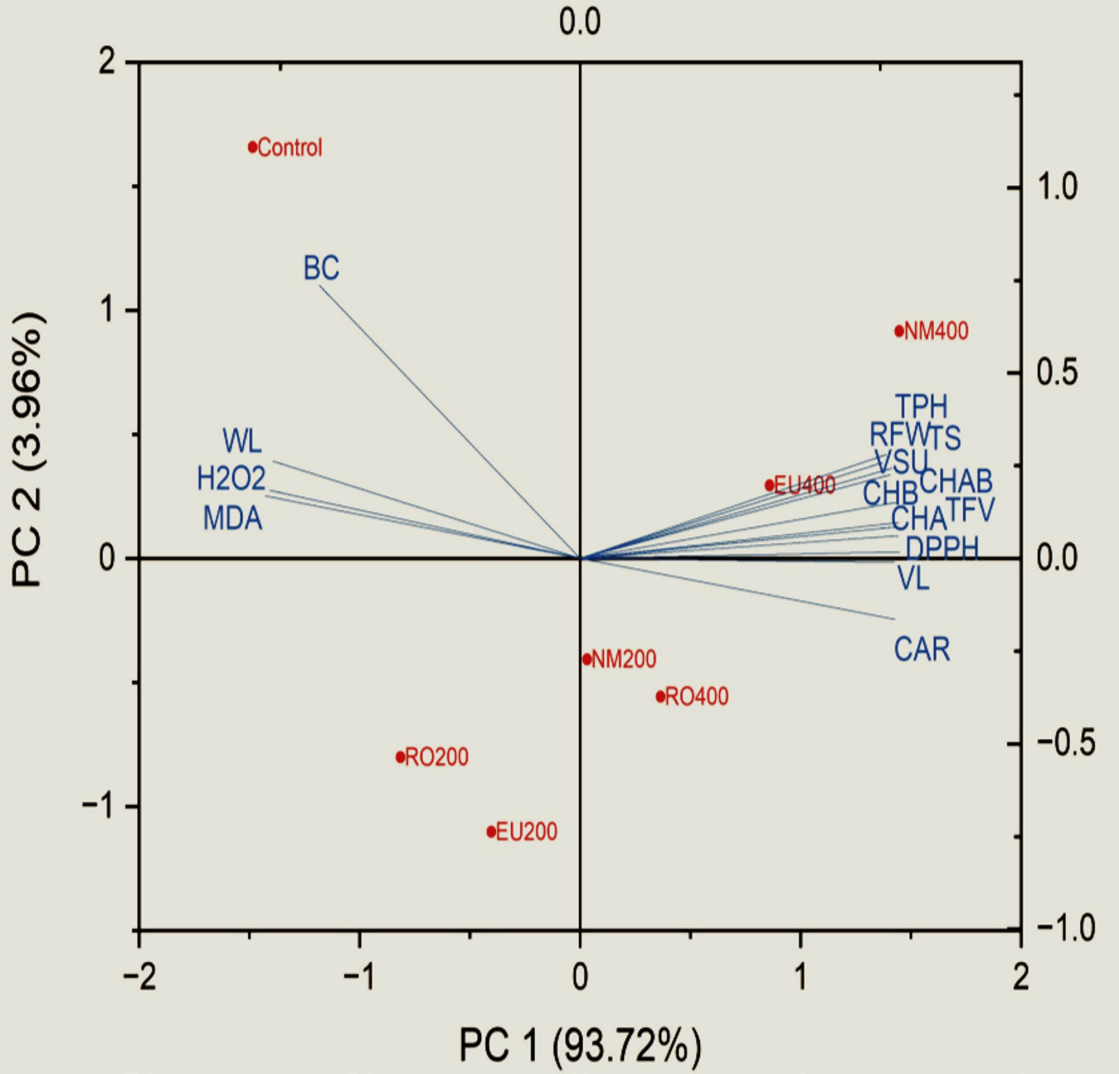
Principal component analysis for solidago cut-flower traits with a relation with different treatments. VL: vase life, VSU: vase solution uptake, WL: water loss, RFW: relative fresh weight, CHA: chlorophyll a, CHB: chlorophyll b, CHAB: chlorophyll ab, CAR: carotenoids, TS: total soluble sugar, TPH: total phenol, TFV: total flavonoids, BC: bacterial count, H_2_O_2_: Hydrogen peroxide, MDA: Malondialdehyde

PCA of cut-flower traits and vase life characteristics of solidago showed two significant components that explained more than 97% of the variability (Fig. 10). The first factor PC1 explained ∼93% of variance and was correlated negatively with water loss, H_2_O_2_ and MDA and positively correlated with vase solution uptake, relative fresh weight, chlorophyll *a*, chlorophyll *b*, total chlorophyll, carotenoids, total soluble sugar, total phenol, total flavonoids. Dominant mechanism influence of PC1 indicates a central biological pathway of oxidative stress governs treatment responses. Thus, variables like H₂O₂/MDA and DPPH are critical biomarkers. NM400 likely enhances vase life, photosynthesis, and antioxidant activity. The second sector PC2 explained ∼3% of the variability and was correlated with the bacterial count. NM400 showed high positive tendency with various treats that positively enhance flower longevity and quality. Whereas, control showed the same tendency of bacterial count, water loss, H_2_O_2_ and MDA content as they all gave negative correlation with PC1. NM200, RO400 and EU200 form a distinct cluster linked to stress-response pigments (carotenoids).

## Discussion

Essential oils are safe and nature-friendly compounds that can be obtained from aromatic plant organs, i.e. flowers, seeds, fruits, fruit peels, leaves, stems, barks, wood and roots. Many essential oils were widely tried out, for example *Apiaceae*, *Lamiaceae* and other plant families to extend the vase life of cut-flowers, i.e., gerbera, gladiolus, rose, chrysanthemum, lisianthus, alstroemeria and carnation [5, 50–59]. Additionally, reduced water intake, carbohydrate creation restriction, vascular clogging, and ethylene production can reduce cut-flower vase life and accelerate dehydration after harvesting [60]– [61]. Essential oils and related compounds used as a protective action against various stress conditions may influence cut-flowers’ vase life. Because of the wide range of ingredients, essential oils appear to have no exact cellular objectives or specific mode of action [59]. Our results support the attribute of using essential oils for extending vase life as has been observed here on solidago cut flowers.

After cutting, the original weight of cut-flowers decreases due to the loss of water uptake and increasing water loss through transpiration [62, 63]. The positive effect of essential oil treatments on the relative fresh weight was also reported in cut-flowers of gerbera [54], Chrysanthemum [64], Gladiolus [65] and Carnation [51, 53]. The present results showed that NM400 treatment significantly increased relative fresh weight, as compared to the control. The relative fresh weight of cut-flowers is an indicator of flower senescence; flower senescence is linked with the capacity for water uptake [66]. On the other hand, the decrease of relative flower weight may occur because of a deficiency of water uptake or an increase of water loss [67]. The increase in flower relative fresh weight with NM400 treatment could be due to facilitating water uptake and reducing the blockage of the vascular system. This mildness of water flow postpones the critical rise of respiration and ethylene production and thus, the freshness of flowers would be maintained longer [68].

The enhancement of water uptake is followed by loss of relative fresh weight that consecutively promotes carbohydrate assimilation in the cells of the petals providing the required energy for sustenance and respiration [69]. Restriction of stomatal opening in separated *Vicia faba* L. leaves occurred when exposed to aromatic organic compounds from *Prinsepia utilis* L. Stomatal closure by essential oils is associated with their potential to prevent K^+^ inflow to the guard cells [70]. It is possible that eucalyptus essential oil might have decreased water loss by controlling the stomatal aperture and accordingly aided in maintaining fresh weight [62, 71].

Vase solution uptake and water loss of cut-flowers are the two foremost markers of vase life [72]. Essential oils were able to inhibit excessive water loss resulting from the control treatment. The use of NM400 showed a significant reduction in water loss. Rapid water loss does highly cause a reduction in water status and flower quality and thus a rapid decline in vase life duration [72].

In agreement with our results, the use of clove oil at 200 mg L^–1^ had the highest chlorophyll and carotenoids content in carnation cv. ‘Cinderella’ [64]. Also, clove oil on lily cv. ‘Sorbonne’, ‘Zambesi’ and ‘Caesars’ cut-flowers showed similar results [73]. In other studies, dill oil on *Helianthus annuus* [74] and geranium, eucalyptus and Myrtus oils on chrysanthemum [50] were found also to promote chlorophyll and carotenoids. Also, it was shown that tea essential oil on chrysanthemum raises the composition of carotene pigments in petals [75]. The abovementioned reported results including results obtained here imply the effectiveness of several natural constituents on the reservation of cut-flowers’ colorants.

The relation explained clearly that higher antioxidant enzyme function has been suggested to scavenge reactive oxygen species (ROS) to alleviate oxidative stress [76–79]. Likewise, it was noticed that higher antioxidant activities of enzymes were authenticated with alleviating causal of oxidative stress [80–84]. Plants defend themselves from the harmful influences of oxidative stress via different resistance mechanisms, involving the enzymatic antioxidant defense system such as polyphenol oxidase, peroxidase, catalase and superoxide dismutase [85–88]. Antioxidant mechanisms approach by suspending or preventing oxidative demolition via its capability to block free radicals [89]– [90]. Phenolic acids, polyphenols and flavonoids scavenge ROS formed in stress circumstances such as oxidation products of phospholipids and thus stop the oxidative processes that cause deterioration and ageing in plants [91–93]. High levels of phenolic compounds support antioxidant defense mechanisms in plants, improving their ability to tolerate stress [94, 95]. Phenolic constituents work as antioxidants via giving hydrogen or electrons alongside via their steadiness as radical mediates [96–98].

Oxidative stress through petal ageing has been explored in cut-flower like rose and dianthus which can be restored by the addition of antioxidants in a preservative solution [86, 99]. When plant extracts are enriched with antioxidants and polyphenols they tend to regulate ROS [100–103].

Many researchers illustrated that essential oil constituents colligate to the cell wall and respond with enzymes accountable for the production of the cell wall and as a consequence lead to pathogen death [104]. Furthermore, the anti-bacterial characteristics of essential oils due to their lipophilic character that condensed in bacterial membranes leading to energy leakage [105]. The effect of essential oils is considered to be the interruption of the cytoplasmic membrane consistency, disrupting the action of the proton, electron flux, kinetic movement, and aggregation of cell components [106].

Likewise, for chrysanthemum cut-flowers, microbial population growth in the vase solution supplemented with essential oils was lower than in untreated one and other supplements [64]. Thus, the anti-bacterial actions of essential oils of aromatic plants could be associated to the enhanced carnation cut-flower shelf life. The reduction of microbial activity has been indicated by Amin and El Sayed [107].

One important factor affecting cut-flowers shelf life and decreasing water relations is microbial invasion in the vase preservative solution. Microorganisms also produce internal ethylene and poisonous substances, causing stem blockage and accelerated aging of flower petals. When microbes increase in the vase solution, hydraulic conductivity in cut-flower stems is reduced [108]. Vase life can be prolonged and bacterial growth and development effectively reduced by using essential oils as antimicrobial agents in the vase preservation solution [109]– [110]. Reduction of water flow caused by xylem obstruction, a restricting variable in the extension of cut-flower vase life and demonstration of early fading of flowers and foliage of the cut-flowers [17]. The cut-flowers would lose their cell turgidity due to the obstruction, decreasing the shelf life of the carnation cut-flowers [53] as has been demonstrated and proven in this research work.

## Conclusions

Applications of eco-friendly tools enhance the ability to use and develop the floriculture market and increases customer satisfaction with long-lasting cut-flowers. Based on the findings of this study, untreated solidago cut-flowers exhibited a shorter vase life; while treated spikes with different essential oils showed a longer vase life, almost double. Neem essential oil at 400 mg L^–1^ enhances water status and maintains relative fresh weight, protects chlorophyll content and improves phenolic and flavonoid content, in addition to the reduction in hydrogen peroxide and malondialdehyde content. As well, a reduction in microbial activity and reduction of vascular blockage due to application of essentials oils resulted in improved postharvest attributes and prolonged vase life of solidago cut-flowers. Essential oils, specifically, neem oil, are regarded more inexpensive than several other additives and environmentally safe therefore it could be utilized in holding solutions as an alternative. However, future investigations are needed to assess the photostability and compatibility of neem oil with commercial preservatives in an attempt to boost its efficiency for prolonging the vase life of cut-flowers.

## Data Availability

Data availability The datasets used and/or analyzed during the present investigation available from the corresponding author on reasonable request.
